# Effective Transport Recovery of Palladium(II) from Hydrochloric Acid Solutions Using Polymer Inclusion Membrane with Tetrabutylammonium Bromide

**DOI:** 10.3390/molecules29133009

**Published:** 2024-06-25

**Authors:** Beata Pospiech

**Affiliations:** Department of Materials Engineering, Czestochowa University of Technology, 19 Armii Krajowej Ave., 42-200 Czestochowa, Poland; beata.pospiech@pcz.pl

**Keywords:** polymer inclusion membranes (PIMs), palladium(II), tetrabutylammonium bromide (TBAB), separation processes

## Abstract

This article reports on the extraction of palladium(II) from hydrochloric acid (HCl) solutions using polymer inclusion membranes (PIMs) containing tetrabutylammonium bromide (TBAB) as the ion carrier. The membranes were based on cellulose triacetate (CTA) as the polymer support. The main aim of this study is to determine the possibility of TBAB’s application as the effective ion carrier/extractant of Pd(II) from hydrochloric acid solutions. At first, the effect of the hydrochloric acid concentration in the aqueous phase on palladium(II) extraction was investigated. Next, cellulose triacetate membranes with TBAB as the carrier were prepared and applied for the recovery of Pd(II) from HCl solutions. As a result of the investigations, the optimal composition of the receiving phase was determined to be 0.5 M thiourea in 0.1 M hydrochloric acid. The effect of the acid concentration in the source phase was investigated. The results show a linear decrease in the permeability coefficient and initial flux of palladium(II) with an increase in the hydrochloric acid concentration in the source phase. The separation of Pd(II) from Pt(IV) from the hydrochloric acid solution was also studied. The transport rate of Pd(II) was higher than Pt(IV). The separation coefficient S_Pd/Pt_ was 1.3. The results show that transport through PIMs with TBAB can be used as an effective method to recover Pd(II) from hydrochloric acid, especially at a low concentration of this acid.

## 1. Introduction

Polymer inclusion membranes (PIMs) containing a polymer (e.g., cellulose triacetate (CTA), poly(vinyl chloride) PVC)), plasticizer (e.g., nitrophenyl ethers), and an ion carrier (e.g., a commercial extractant) have been designed for the transport and selective separation of many metal ions from aqueous solutions [1,2,3,4,5,6,7]. Membrane processes are very often applied for the separation of different substances such as metal ions [1,2,3,4,8,9,10], as well as small molecules (e.g., antibiotics) [5], inorganic anions and organic substances [8].

The composition of the PIM plays an important role in the efficiency of the transport of various chemical particles and ions through the PIM [1,2,3,4,5,7,8,9,10,11,12,13,14,15,16]. The base polymer provides the PIM with mechanical strength [1,2], while plasticizers make the polymers softer and more flexible, as well as more stable under different experimental conditions [8]. Moreover, a plasticizer is used as a solvent of the ion carrier in order to make the transported species more soluble in the membrane liquid phase [17]. Polymer inclusion membranes constitute a type of liquid membranes. The mechanism of transport for metal ions has been described by many researchers [18,19].

PIMs have already been shown to be applicable in the separation of platinum group metals (PGMs) [20,21,22,23]. Secondary raw materials in the form of spent catalysts are an important source of precious metals [20,21,22,23,24,25]. Hanada et al. [22] studied the transport of rhodium(III) and iron(III) across PIMs with (dodecyl)phosphonium chloride (P_88812_Cl) as the metal ion carrier. The metal ions were transported from 0.1 HCl mol·dm^−3^ into 0.1 mol·dm^−3^ HCl containing 4.9 mol·dm^−3^ NH_4_Cl. They reported that more than 70% Rh(III) was recovered and the transport of Fe(III) was not observed. Fajar et al. [23] investigated the recovery of platinum (IV), palladium(II) and rhodium(III) from a spent automotive catalyst using polymer inclusion membranes containing the ionic liquid trioctyl (dodecyl) phosphonium chloride (P_88812_-Cl). They studied the selective transport of PGMs from a SAC (spent automotive catalyst) leachate solution using PIMs. They reported that more than 90% of Pt(IV) and Pd(II) from the SAC extraction solutions could be recovered. Moreover, they observed that polymer membranes demonstrated excellent durability over a 10-day exposure to various concentrated inorganic acids.

As can be seen, ionic liquids (ILs) are very often used as the carriers of metal ions for membrane synthesis [8,9,10,11,12,13,21,22,23,24] and as the extractants of PGMs [10,26,27,28,29,30,31,32] in the conventional solvent extraction process. However, there have been no reports to date on palladium(II) transport using PIMs with TBAB. The preliminary studies of the solvent extraction of Pd(II) from hydrochloric acid solutions showed that this compound is a very good extractant of this metal ion. It is worth mentioning that in the transport of the metal ion through PIMs, extraction and re-extraction occur at the same time. Thus, the efficiency of the re-extraction is equally important as that of the extraction.

The main aim of this study is to investigate the kinetic transport of Pd(II) from aqueous solutions through PIMs with TBAB as the ion carrier. The second important aim is to determine the appropriate conditions for high-efficiency palladium extraction from a hydrochloric acid solution. To achieve this goal, the effect of the source and receiving phases on the efficiency of metal recovery across PIMs was investigated. The results should be of valuable interest for the development of a new method for the recovery of Pd(II) through PIMs with TBAB.

## 2. Results and Discussion

### 2.1. Solvent Extraction of Pd(II) by TBAB

**Figure 1:**
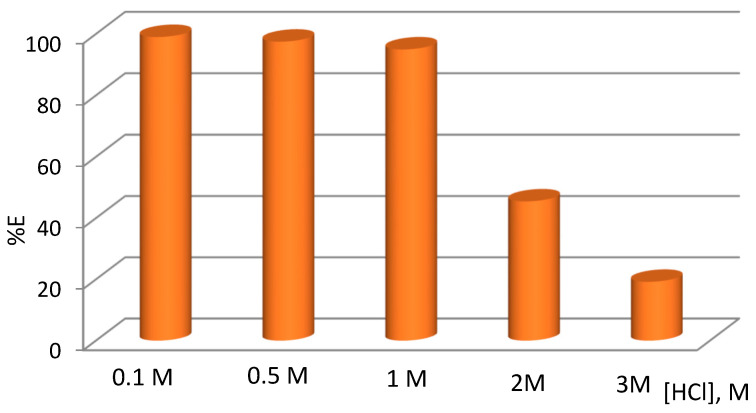
Effect of HCl concentration on extraction efficiency of Pd(II) with 0.1 M TBAB in toluene.

Hydrochloric acid is very often used as the leaching agent of palladium from secondary sources of precious metals (e.g., spent catalysts). Therefore, the research focused on HCl solutions. The chemistry of palladium chlorocomplexes is very important, as it is crucial in explaining the mechanism of extraction and membrane transport [28]. In aqueous chloride solutions, palladium forms chlorocomplexes depending on the HCl concentration. According to the data in the literature, the main complex form of Pd(II) in a chloride medium containing 0.1 mol·dm^−3^ and higher chloride ion concentration is PdCl_4_^2−^ [31,32]. In order to better understand the transport behavior of Pd(II) through the PIM with TBAB as an ion carrier, it was necessary to perform solvent extraction studies with this compound as the extractant. The solvent extraction of Pd(II) from the HCl solutions was carried out. The concentration of acid in the source phase varied from 0.1 to 3 mol·dm^−3^. Figure 1 shows the effect of the HCl concentration on the extraction efficiency (%E) of Pd(II) with 0.1 M TBAB in toluene. As can be seen, the %E of Pd(II) decreased with the concentration of HCl. The extraction efficiency was very high and reached a maximum of 98.9% at 0.1 mol·dm^−3^ HCl. At a higher HCl concentration, the extraction efficiency was much lower, e.g., 17.9% at 3 mol·dm^−3^ HCl. This decrease can be explained in terms of competition between PdCl_4_^2−^ and HCl to react with the ion carrier—TBAB. This phenomenon can be explained by the co-extraction of hydrochloric acid at a high concentration of this acid in the source phase. A similar dependence was observed for the transport of Pd(II) through the PIM with [A336][TS] [21]. The predominant form of Pd(II) both in 0.1 M and 3 mol·dm^−3^ HCl is PdCl_4_^2−^ [32]. The results suggest that the extraction of Pd(II) with TBAB proceeds according to the anion-exchange mechanism, similar to the extraction with Cyphos IL 104 [31,32], where the anionic group of the ionic liquid is replaced by the chlorocomplex anion [28]. Cieszynska and Wieczorek [29] reported that palladium(II) is extracted from 0.1 mol·dm^−3^ HCl to the organic phase of the molar ratio of 1:2. Thus, it can be concluded that palladium(II) is extracted to the organic phase according to the following equation:

(1) $$2[(\mathrm{Bu}{)}_{4}N{{]}^{+}}_{\left(\mathrm{org}\right)}+{{{\mathrm{PdCl}}_{4}}^{2-}}_{\left(\mathrm{aq}.\right)}↔{\left[{\left((\mathrm{Bu}{)}_{4}N\right)}_{2}{\mathrm{PdCl}}_{4}\right]}_{\left(\mathrm{org}\right)}$$

The anionic metal chlorocomplexes of palladium are extracted from the aqueous to the organic phase by an anion-exchange mechanism. This mechanism is similar to the extraction of Pd(II) with other ionic liquids such as quaternary ammonium or phosphonium salts [28,29].

### 2.2. Transport Kinetics of Pd(II) from Hydrochloric Acid Solutions across PIM with TBAB

The study of the transport kinetics of Pd(II) through PIMs with TBAB can provide important knowledge concerning the efficiency of this process. Considering the strong complexing properties of TBAB when used as the extractant, high values for the initial flux (Ji) and the permeability coefficient (P) should be expected.

In this experiment, the composition of the membrane was as follows: 47.1% *w*/*w* CTA, 32.7% *w*/*w* NPOE and 20.2% *w*/*w* TBAB. The concentration of TBAB was 1.5 mol·dm^−3^ (based on the volume of plasticizer). The PIM was prepared according to the procedure described in previous papers [9,11,15]. Pd(II) was transported from the 0.1 mol·dm^−3^ HCl solution into 0.5 mol·dm^−3^ thiourea in 0.1 mol·dm^−3^ HCl as the receiving phase. This receiving phase was chosen based on previous results of palladium(II) transport through PIMs with similar ion carriers [21]. There, the thiourea solution in hydrochloric acid proved to be an excellent receiving phase for Pd(II).

**Figure 2:**
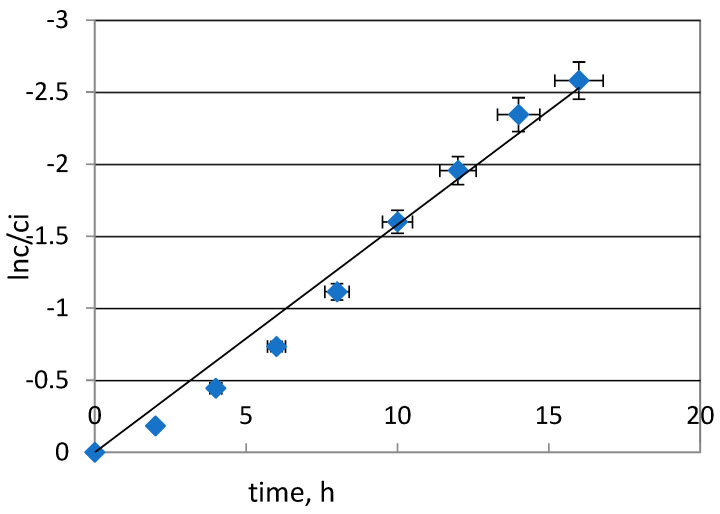
Relationship of ln(*c*/*c_i_*) vs. time for Pd(II) transport through PIMs; source phase: 0.001 mol·dm^−3^ Pd(II) in 0.1 mol·dm^−3^ HCl, receiving phase: 0.5 M thiourea in 0.1 mol·dm^−3^ HCl.

To calculate the k value (rate constant), a plot of ln(*c*/*c_i_*) vs. time was prepared. Figure 2 shows a graph of the dependence of ln(*c*/*c_i_*) vs. time for Pd(II) transport. As can be seen from this figure, the relationship of ln(*c*/*ci*) vs. time is linear. Thus, it can be concluded that the transport of palladium(II) was described by a first-order reaction:

(2) $$\ln\left(\frac{c}{c_{i}}\right)=-kt$$

where

*c*—palladium(II) concentration (mol/dm^3^) in the source phase at a given time,

*c_i_*—palladium(II) ion concentration in the source phase (mol/dm^3^),

*t*—time (s),

*k*—rate constant (s^−1^).

Kinetic parameters such as the permeability coefficient (*P*) and initial flux (*J_i_*) were calculated according to the equations presented in previous works [9,11,15]. The results are presented in Table 1. The rate constant of this transport process was 0.158 h^−1^. The recovery factor (RF) of Pd(II) was 92.4%. As can be seen, the transport of Pd(II) across PIMs with TBAB is very effective and the results can be recognized as promising. For comparison, the use of a magnetic sorbent containing an organophosphate extractant resulted in the sorption efficiency of palladium(II) reaching 71% in one cycle. Yudaev et al. [33] reported that only when the sorbent was treated with 5 M hydrochloric acid was the palladium completely extracted from the sorbent.

The permeability coefficient (*P*) was calculated according to the equations presented in previous works [9,11,15]. The permeability coefficient (*P*) can be calculated as follows:

(3) $$P=\frac{V}{A}k,$$

where *V*—volume of the aqueous source phase, and *A*—an area of the effective membrane.

**Figure 3:**
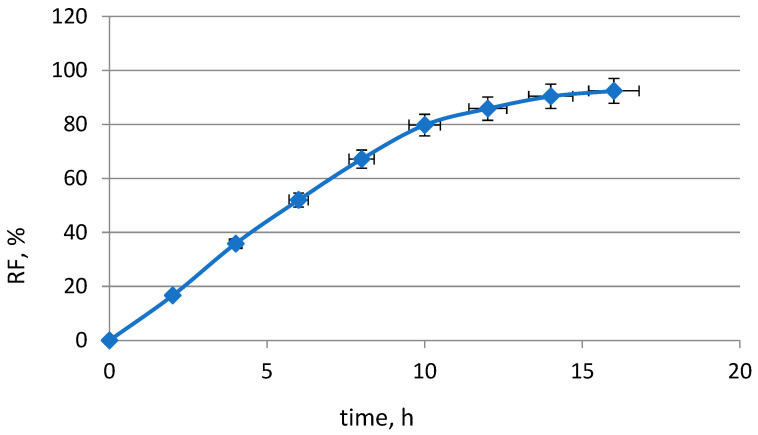
Recovery factor of Pd(II). Conditions as in the Figure 2.

Figure 3 shows the recovery factor of Pd(II), which was calculated as

(4) $$RF=\frac{c_{i}-c}{c_{i}}\cdot 100\%$$

### 2.3. Effect of Thiourea Concentration in Stripping Phase

**Figure 4:**
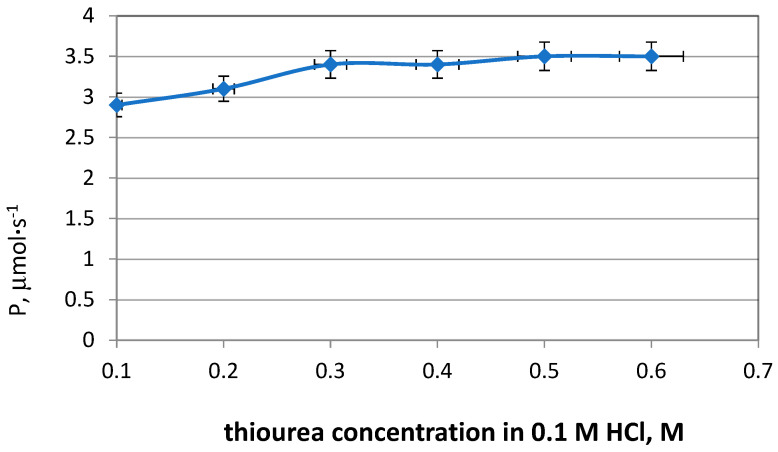
Effect of thiourea concentration in 0.1 mol·dm^−3^ HCl as receiving phase on permeability coefficient (P). Source phase: 0.001 mol·dm^−3^ Pd(II) in 0.1 mol·dm^−3^ HCl.

The type of receiving phase is a very important factor in determining the efficiency of the extraction of metals both during solvent extraction and during transport through polymer inclusion membranes. A review of the literature and previous research has shown that a solution of urea in HCl can be successfully used as a receiving phase for palladium. The aim of the next series of experiments was to investigate the effect of the thiourea concentration in the receiving phase on the transport rate of Pd(II). The concentration of thiourea in 0.1 mol·dm^−3^ HCl varied from 0.1 to 0.6 mol·dm^−3^. The permeability coefficient of Pd(II) increased with an increase in the thiourea concentration in the receiving phase and, as can be observed from Figure 4, the highest value was obtained for 0.5 mol·dm^−3^ thiourea in 0.1 mol·dm^−3^ HCl. The very good properties of the solution of thiourea as the stripping phase are also confirmed by numerous studies by other researchers [32,34].

### 2.4. Selectivity Study

**Figure 5:**
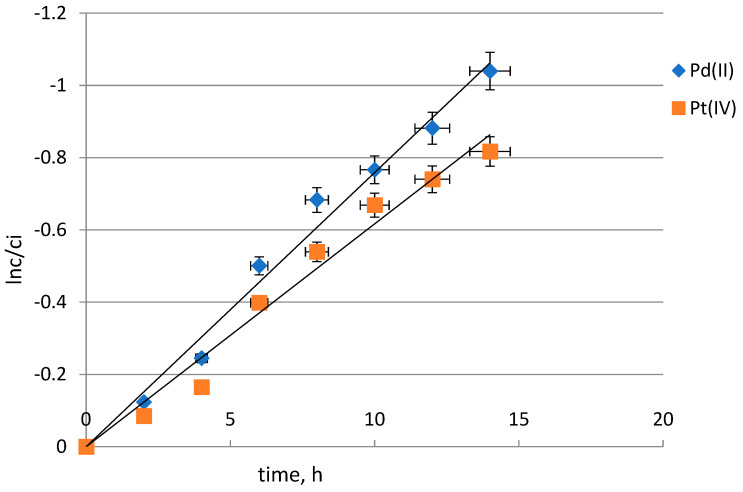
Relationship of ln(*c*/*c_i_*) vs. time for Pd(II) and Pt(IV) transport through PIMs; source phase: 0.001 mol·dm^−3^ Pd(II) and 0.001 mol·dm^−3^ Pt(IV) in 0.1 mol·dm^−3^ HCl, receiving phase: 0.5 mol·dm^−3^ thiourea in 0.1 mol·dm^−3^ HCl.

**Figure 6:**
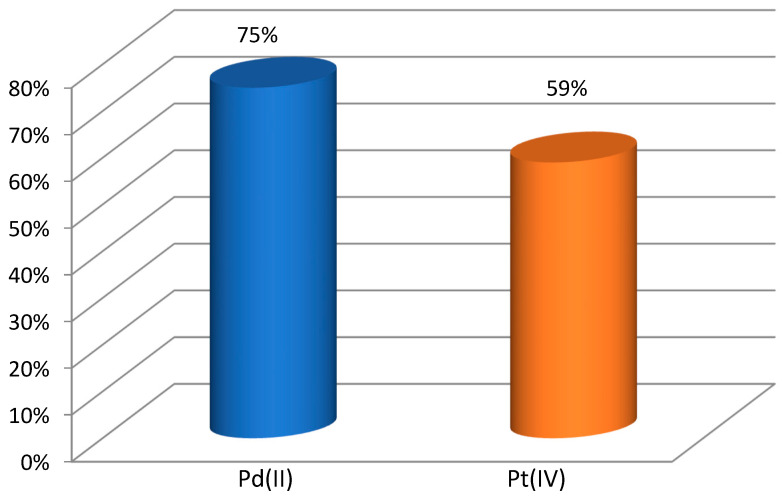
Recovery factor of Pd(II) and Pt(IV). Conditions as in Figure 4.

The selectivity of the extraction of palladium(II) towards platinum(IV) from 0.1 mol·dm^−3^ HCl was also studied. Figure 5 presents a graph of the dependence of ln(*c*/*c_i_*) vs. time for Pd(II) and Pt(IV) across PIMs with TBAB. For the receiving phase, 0.5 mol·dm^−3^ thiourea in 0.1 mol·dm^−3^ HCl was used. The values of the transport rate, permeability coefficient, selectivity order and selectivity ratio for the competitive transport of the metal ions under investigation are summarized in Table 2. In the presence of Pt(IV), the transport rate of Pd(II) differed from that in the single metal system. The results show the permeability coefficients of Pd(II) and Pt(IV) across PIMs with a TBAB decrease in the sequence Pd(II) > Pt(IV). The recovery factors (%) of Pd(II) and Pt(IV) are shown in Figure 6. The highest recovery factor was obtained for Pd(II) and was over 75%. According to the data in the literature, Pt(IV), like Pd(II), occurs as the anionic complex PtCl_6_^2−^. As can be seen, despite everything, palladium(II) was transported more efficiently into the receiving phase. Yudaev et al. [35] reported that ILs increase the efficiency and selectivity of metal extraction by several orders of magnitude, and they can be isolated by re-extraction and reused for other separation processes such as solvent extraction, adsorption, and membrane separation.

### 2.5. Stability of PIMs with TBAB

The stability of the PIMs containing 47.1% *w*/*w* CTA, 32.7% *w*/*w* NPOE and 20.2% *w*/*w* TBAB was evaluated on the basis of the initial flux values obtained from three sequential experiments in which the membrane was used under the following experimental conditions: 0.001 M Pd(II) in 0.1 M HCl as the source phase and 0.5 M thiourea in 0.1 M HCl as the receiving phase. The PIM was removed from the cell and washed in deionized water. Three experiments were repeated using the same membrane. The initial flux of Pd(II) varied after five cycles of 16 h each. In Table 3, the variation in the initial flux of Pd(II) for all the experiments is shown. As can be seen, the initial flux decreased from 3.5 to 2.8 μmol∙s^−1^∙m^−2^. The changes indicate that this membrane with TBAB is relatively stable. It can be concluded that PIMs containing this ion carrier can be used for the recovery of Pd(II) from acidic solutions, i.e., from the leach liquor of spent automotive catalysts.

The initial flux (*Ji*) was determined as [9,11,15]

(5) $$J_{i}=P\cdot c_{i}$$

## 3. Materials and Methods

Reactions were conducted using two kinds of reagents. The first group was inorganic compounds, as follows: PdCl_2_ in purity of 99% (purchased in Sigma Aldrich, St. Louis, MO, USA), 30% H_2_PtCl_6_ and HCl (delivered by POCh, Gliwica, Poland). These compounds were applied in form of aqueous solution prepared with deionized water. In the second group, the organic reagents cellulose triacetate (CTA) (Sigma-Aldrich, St. Louis, MO, USA), 2-Nitrophenyl octyl ether (NPOE) (Sigma-Aldrich, St. Louis, MO, USA), tetrabutylammonium bromide (TBAB) (ACROS, Bridgewater, NJ, USA) (purity ≥ 99.0%), dichloromethane (P.P.H. STANLAB Sp. J., Lublin, Poland) and thiourea (POCh, Gliwice, Poland) with analytical purity were applied without further purification.

The polymer inclusion membranes (PIMs) were synthesized using a technique similar to that described in the following papers [9,11,15]. During the current study, the PIMs using CTA as the base polymer were produced using the method presented in [9,11,15]. The TBAB and NPOE were used as the ion carrier of Pd(II) and the plasticizer, respectively. The polymer membranes were prepared with the following components and stoichiometry: 47.1% *w*/*w* CTA, 32.7% *w*/*w* NPOE and 20.2% *w*/*w* TBAB (Figure 1). The ion carrier was made for a concentration of TBAB of 1.5 mol·dm^−3^.

The transport experiments were carried out according to the procedure described in earlier papers [9,11,15]. The membrane segments were used for the removal of Pd(II) during transport from hydrochloric acid, which was applied as the source across the PIM into the destination stage using a peristaltic pump (PP1B-05A, Zalimp, Warszawa, Poland).

The membranes separated the source and receiving phases. The initial volume of the source and destination phases equaled 100 cm^3^. The concentration of the Pd(II) ion in the source phase was 0.001 mol·dm^−3^ soluted in hydrochloric acid. The receiving phase was 0.5 mol·dm^−3^ thiourea soluted in 0.1 mol·dm^−3^ hydrochloric acid. The area of the PIM equaled 12.56 cm^2^. The magnetic stirrers were used to stir the source (containing Pd(II)) and the receiving phases. A plasma emission spectrometer MP-AES 4200 (Agilent, Santa Clara, CA, USA) was applied to reveal a concentration of palladium(II).

The same volumes of aqueous and organic phases (phase volume ratio O/A = 1) were shaken for 15 min at 22 ± 2 °C. The aqueous phase was the HCl solution containing 0.001 mol·dm^−3^ Pd(II), while the organic phase contained 0.1 mol·dm^−3^ TBAB in toluene. During the next step, the aqueous phase was separated from the organic phase.

### 3.1. Reagents

#### 3.1.1. Inorganic Reagents

The inorganic reagents were palladium(II) chloride, PdCl_2_ (purity = 99%), supplied by Sigma Aldrich (St. Louis, MO, USA); chloroplatinic acid, (30% H_2_PtCl_6_, pure); and hydrochloric acid (HCl) (POCh, Gliwice, Poland). The aqueous solutions were prepared with deionized water.

#### 3.1.2. Organic Reagents

The organic reagents were cellulose triacetate (CTA) (Sigma-Aldrich, St. Louis, MO, USA), 2-Nitrophenyl octyl ether (NPOE) (Sigma-Aldrich, St. Louis, MO, USA), tetrabutylammonium bromide (TBAB) (ACROS, Bridgewater, NJ, USA) (purity ≥ 99.0%), dichloromethane (P.P.H. STANLAB Sp. J., Lublin, Poland) and thiourea (POCh, Gliwice, Poland) and were of analytical grade and used without further purification.

### 3.2. Synthesis of Polymer Inclusion Membranes (PIMs)

The PIMs were prepared similarly to the method reported in earlier papers [9,11,15]. In this study, PIMs using CTA as the base polymer were prepared as previously described [9,11,15]. TBAB was used as the ion carrier of Pd(II) and NPOE was the plasticizer. The components of the polymer membrane were the following: 47.1% *w*/*w* CTA, 32.7% *w*/*w* NPOE and 20.2% *w*/*w* TBAB. The concentration of TBAB used as the ion carrier was 1.5 mol·dm^−3^ (based on the volume of plasticizer). In this study, PIMs using CTA as the base polymer, TBAB as the ion carrier and NPOE used as the plasticizer were prepared as previously described [9,11,15].

### 3.3. Transport of Metal Ions Experiments

The transport experiments were conducted as reported in earlier papers [9,11,15]. The membrane module was used for the transport of Pd(II) from hydrochloric acid, which was used as the source phase across the PIM into the receiving phase. The PIM separated the source and receiving phases. Both of the phases were pumped with a peristaltic pump (PP1B-05A, Zalimp, Poland). The volume of the source and receiving phases was 100 cm^3^. The source phase contained 0.001 mol·dm^−3^ Pd(II) in a hydrochloric acid solution, while 0.5 mol·dm^−3^ thiourea in 0.1 mol·dm^−3^ hydrochloric acid was used as the receiving phase. The PIM area was 12.56 cm^2^. The source phase containing Pd(II) and the receiving phase were stirred with magnetic stirrers. The concentration of palladium(II) was analyzed using a plasma emission spectrometer MP-AES 4200 (Agilent). The essential advantage of MP-AES over flame AAS is it safety. Plasma can be considered to be a partially ionized gas containing electrons, ions, neutral species, excited particles, and photons. The application of AES is very useful and practical.

### 3.4. Solvent Extraction of Pd(II)

Equal volumes of aqueous and organic phases (phase volume ratio O/A = 1) were shaken for 15 min at 22 ± 2 °C 0.1 mol·dm^−3^. The aqueous phase was the HCl solution containing 0.001 mol·dm^−3^ Pd(II), while the organic phase contained 0.1 mol·dm^−3^ TBAB in toluene. Next, the aqueous phase was separated from the organic phase. The Pd(II) concentration in the aqueous phase was analyzed using a plasma emission spectrometer MP-AES 4200 (Agilent).

## 4. Conclusions

The present paper described the use of a CTA-based PIM containing TBAB as the ion carrier for the efficient extraction of Pd(II) from hydrochloric acid solutions. The low concentration of HCl enables the effective transport of Pd(III) from the source phase into 0.5 mol·dm^−3^ thiourea and 0.1 mol·dm^−3^ HCl as the receiving phase. This method can be used to recover Pd(II) from hydrochloric acid solutions. The membrane extraction of Pd(II) is faster and more efficient than Pt(IV). In the future, it will be necessary to study the separation of precious metals (Pd(II), Pt(IV), Au(III)) from real solutions after leaching spent catalysts.

## Figures and Tables

**Table 1:** Kinetic parameters for transport of Pd(II). Condition as in Figure 3.

Kinetic Parameters	Values
Rate constant (*k*), h^−1^	0.158
Permeability coefficient (*P*), μmol∙s^−1^	3.50

**Table 2:** Kinetic parameters for transport of Pd(II) and Pt(IV). Condition as in Figure 5.

Kinetic Parameters	Pd(II)	Pt(IV)	Selectivity Order and Selectivity Coefficient
Rate constant (*k*), h^−^	0.079	0.06	Pd(II) > Pt(IV)
Permeability coefficient (*P*), μmol∙s^−1^	1.75	1.32	S_Pd/Pt_ = 1.3

**Table 3:** Effect of number of cycles for Pd(II) transport across PIMs on permeability coefficient. Conditions as in Figure 3.

Cycle Number	Initial Flux, μmol∙s^−1^∙m^−2^
1	3.5
2	3.1
3	2.8

## Data Availability

Data are contained within the article.

## References

[1] Kolev S.D., Sakai Y., Cattrall R.W., Paimin R., Potter I.D. (2000). Theoretical and experimental study of palladium(II) extraction from hydrochloric acid solutions into Aliquat 336/PVC membranes. Anal. Chim. Acta.

[2] Kozlowski C.A., Walkowiak W. (2005). Applicability of liquid membranes in chromium(VI) transport with amines as ion carriers. J. Membr. Sci..

[3] Macías M., Rodríguez de San Miguel E. (2023). On the Use of polymer inclusion membranes for the selective separation of Pb(II), Cd(II), and Zn(II) from seawater. Membranes.

[4] Eyupoglu V., Unal A. (2023). The extraction and the removal of Cd(II) using polymer inclusion membrane containing symmetric room temperature ionic liquid as ion carrier. J. Environ. Chem. Eng..

[5] Garcia-Rodriguez A., Fontas C., Matamoros V., Almeida M.I.G.S., Cattrall R.W., Kolev S.D. (2016). Development of a polymer inclusion membrane-based passive sampler for monitoring of sulfamethoxazole in natural waters. Minimizing the effect of the flow pattern of the aquatic system. Microchem. J..

[6] Regel-Rosocka M., Alguacil F.J. (2013). Recent trends in metal extraction. Rev. Metal..

[7] Dudek S., Kołodyńska D. (2022). Arsenate removal on the ion exchanger modified with cerium(III) ions. Physicochem. Probl. Miner. Process..

[8] Vazguez M.I., Romero V., Fontas C., Antico E., Benavente J. (2014). Polymer inclusion membranes (PIMs) with the ionic liquid (IL) Aliquat 336 as extractant: Effect of base polymer and IL concentration on their physical-chemical and elastic characteristics. J. Membr. Sci..

[9] Pospiech B. (2015). Studies on extraction and permeation of cadmium(II) using Cyphos IL 104 as selective extractant and ion carrier. Hydrometallurgy.

[10] Wiecka Z., Rzelewska-Piekut M., Reis M.T.A., Ismael M.R.C., Wieszczycka K., Regel-Rosocka M. (2023). Pd(II) and Pt(IV) dispersive or non-dispersive extraction from model and real leach solutions with alkoxyimine-1-propylpyridinium derivatives. Sep. Purif. Technol..

[11] Makowka A., Pospiech B. (2020). Studies on extraction and competitive permeation of cerium(III) and lanthanum(III) using Cyphos IL104 as selective extractant and ion carrier. Sep. Sci. Technol..

[12] Zeng X., Xu L., Deng T., Zhang C., Xu W., Zhang W. (2022). Polymer Inclusion Membranes with P507-TBP Carriers for Lithium Extraction from Brines. Membranes.

[13] Pospiech B., Kujawski W. (2015). Ionic liquids as selective extractants and ion carriers of heavy metal ions from aqueous solutions utilized in extraction and membrane separation. Rev. Chem. Eng..

[14] Nghiem L.D., Mornane P., Potter I.D., Perera J.M., Cattrall R.W., Kolev S.D. (2006). Extraction and transport of metal ions and small organic compounds using polymer inclusion membranes (PIMs). J. Membr. Sci..

[15] Pospiech B. (2022). Separation of Co from Ni and Li from chloride media using polymer inclusion membrane system with thiosalicylate based ionic liquid. Physicochem. Probl. Miner. Process..

[16] Bashiri A., Nikzad A., Maleki R., Asadnia M., Razmjou A. (2022). Rare earth elements recovery using selective membranes via extraction and rejection. Membranes.

[17] Almeida M.I.G.S., Cattrall R.W., Kolev S.D. (2017). Polymer inclusion membranes (PIMs) in chemical analysis—A review. Anal. Chim. Acta.

[18] De San Miguel E.R., Hernández-Andaluz A.M., Bañuelos J.G., Saniger J.M., Aguilar J.C., de Gyves J. (2006). LIX^®^-loaded polymer inclusion membrane for copper(II) transport: 1. Composition–performance relationships through membrane characterization and solubility diagrams. Mater. Sci. Eng. A.

[19] De Gyves J., Hernández-Andaluz A.M., de San Miguel E.R. (2006). LIX^®^-loaded polymer inclusion membrane for copper (II) transport: 2. Optimization of the efficiency factors (permeability, selectivity, and stability) for LIX^®^ 84-I. J. Membr. Sci..

[20] Pospiech B. (2015). Highly efficient facilitated membrane transport of palladium(II) ions from hydrochloric acid solutions through plasticizer membranes with Cyanex 471X. Physicochem. Probl. Miner. Process..

[21] Pospiech B. (2018). Facilitated transport of palladium(II) across polymer inclusion membranes with ammonium ionic liquid as effective carrier. Chem. Pap..

[22] Hanada T., Firmansyah M.L., Yoshida W., Kubota F., Kolev S.D., Goto M. (2020). Transport of rhodium(III) from chloride media across a polymer inclusion membrane containing an ionic liquid metal ion carrier. ACS Omega.

[23] Fajar A.T.N., Hanada T., Goto M. (2021). Recovery of platinum group metals from a spent automotive catalyst using polymer inclusion membranes containing an ionic liquid carrier. J. Membr. Sci..

[24] Bonggotgetsakul Y.Y.N., Cattrall R.W., Kolev S.D. (2015). Extraction of gold(III) from hydrochloric acid solutions with a PVC-based polymer inclusion membrane (PIM) containing Cyphos^®^ IL 104. Membranes.

[25] Saternus M., Fornalczyk A. (2013). Possible ways of refining precious group metals (PGM) obtained from recycling of the used auto catalytic converters. Metalurgija.

[26] Firmansyah M.L., Kubota F., Yoshida W., Goto M. (2019). Application of a novel phosphonium-based ionic liquid to the separation of platinum group metals from automobile catalyst leach liquor. Ind. Eng. Chem. Res..

[27] Nguyen V.T., Lee J.C., Chagnes A., Kim M.S., Jeong J., Cote G. (2016). Highly selective separation of individual Platinum Group Metals (Pd, Pt, Rh) from acidic chloride media using phosphonium-based ionic liquid in aromatic diluent. RSC Adv..

[28] Pianowska K., Kluczka J., Benke G., Goc K., Malarz J., Ochmański M., Leszczyńska-Sejda K. (2023). Solvent extraction as a method of recovery and separation of Platinum Group Metals. Materials.

[29] Cieszyńska A., Wieczorek D. (2020). Efficiency and mechanism of palladium(II) extraction from chloride media with n-hexadecylpiperidinium chloride. J. Sol. Chem..

[30] Regel-Rosocka M., Rzelewska M., Baczynska M., Janus M., Wisniewski M. (2015). Removal of palladium(II) from aqueous chloride solutions with Cyphos phosphonium ionic liquids as metal ion carriers for liquid-liquid extraction and transport across polymer inclusion membranes. Physicochem. Probl. Min. Process..

[31] Cieszynska A., Wisniewski M. (2012). Extractive recovery of palladium(II) from hydrochloric acid solutions with Cyphos IL 104. Hydrometallurgy.

[32] Cieszynska A., Wisniewski M. (2011). Selective extraction of palladium(II) from hydrochloric acid solutions with phosphonium extractants. Sep. Purif. Technol..

[33] Yudaev P., Butorova I., Stepanov G., Chistyakov E. (2022). Extraction of palladium(II) with a magnetic sorbent based on polyvinyl alcohol gel, metallic iron, and an environmentally friendly polydentate phosphazene-containing extractant. Gels.

[34] Sun P.P., Lee M.S. (2011). Separation of Pt(IV) and Pd(II) from the loaded Alamine 336 by stripping. Hydrometallurgy.

[35] Yudaev P.A., Chistyakov E.M. (2022). Ionic liquids as components of systems for metal extraction. ChemEngineering.

